# Use of antimicrobials in pediatric wards of five Brazilian hospitals

**DOI:** 10.1186/s12887-024-04655-9

**Published:** 2024-03-13

**Authors:** Thais de Barros Fernandes, Sheila Feitosa Ramos, Luísa Rodrigues Furtado Leitzke, Ronaldo Gomes Alexandre Júnior, Janaína Morais de Araújo, Alcidésio Sales de Souza Júnior, Alice Ramos Oliveira da Silva, Isabela Heineck, Marta Maria de França Fonteles, Louise E. Bracken, Matthew Peak, Divaldo Pereira de Lyra Junior, Claudia G S Osorio-de-Castro, Elisangela Costa Lima

**Affiliations:** 1 Sergio Arouca National School of Public Health, Oswaldo Cruz Foundation, Rio de Janeiro, 21041-210 Brazil; 2 Health Sciences Graduate Program, Social Pharmacy Teaching and Research Laboratory (LEPFS), Federal University of Sergipe, São Cristóvão, Brazil; 3 Postgraduate Program in Pharmaceutical Services, Faculty of Pharmacy, Federal University of Rio Grande do Sul, Porto Alegre, Brazil; 4 Department of Pharmacy, Faculty of Pharmacy, Dentistry and Nursing, Federal University of Ceará, Fortaleza, Brazil; 5 Antonio Lisboa Mother and Child Hospital, Brasília, Brazil; 6 School of Pharmacy, Federal University of Rio de Janeiro, Rio de Janeiro, Brazil; 7 Paediatric Medicines Research Unit, Institute in the Park, Alder Hey Children’s NHS Foundation Trust, Liverpool, UK; 8 Center for Biological and Health Sciences, Federal University of Western Bahia, Barreiras, Brazil

**Keywords:** Antimicrobial agents, Drug utilization, Off-label use, hospital, Pediatric

## Abstract

The use of antimicrobials (AMs) in pediatric infections is common practice and use may be inappropriate leading to antimicrobial resistance. Off-label AM use is also common in this group and can result in drug-related problems. There is lack of DUR data in Brazil and in Latin America, specially for AM pediatric use. The aim of this study was to describe the utilization of AMs in hospitalized children in five hospitals in Brazil. We conducted an observational study of the utilization of AMs in pediatric wards in hospitals in the states of Ceará (CE), Sergipe (SE), Rio de Janeiro (RJ), Rio Grande do Sul (RS) and the Federal District (DF). Data derived from patient medical records and prescriptions were collected over a six-month period in each hospital. The number of AMs used by each patient was recorded, and AM use was assessed using Days of therapy (DOT) and Length of therapy (LOT) per 1000 patient days according to different patient characteristics. Off-label (OL) use was described according to age. The study analyzed data from 1020 patients. The sex and age distributions were similar across the five hospitals. However, differences were found for comorbidities, history of ICU admission and length of hospital stay. The most common diseases were respiratory tract infections. There were wide variations in DOT/1000PD (278–517) and LOT/1000PD (265–390). AM utilization was highest in the hospital in SE. The consumption of second-generation penicillins and cephalosporins was high. The prevalence of OL use of AMs was higher for patients in the RJ hospital, in infants, in patients who underwent prolonged hospital stays, and in patients who used multiple AMs. The AM that showed the highest prevalence of OL use was azithromycin, in both oral and parenteral formulations. Overall AM use was high and showed differences in each setting, possibly influenced by local characteristics and by prescribing standards adopted by pediatricians.

**Supplementary Information:**

The online version contains supplementary material available at 10.1186/s12887-024-04655-9.

## Background

Infections are among the most frequent childhood ailments. As a result, the use of antimicrobials (AMs) in pediatric patients is common practice [1]. The use of these medicines is even higher in hospitalized pediatric patients. A multicenter study using data from 1278 children admitted to pediatric general medical wards in the United Kingdom, Germany, Australia, Hong Kong and Malaysia found that systemic antibacterials were the most commonly prescribed drug class, accounting for 25% of all prescriptions [2]. Two studies analyzing the consumption of antimicrobials in Europe (32 hospitals) and the United States (51 hospitals) reported that a third of hospitalized children received at least one antimicrobial during their hospital stay [3].

When examining AM utilization among hospitalized children, a number of variables are used for characterization apart from age and sex. Hospitalized children may present not only with infections but several comorbidities which may make treatments more difficult and modulate dose and treatment interval (ref). Ascertaining disease severity may also be challenging. A possible way to gage this is by measuring the length of stay, and/or time spent in an Intensive Care Unit (ICU). Additional variables to study are number and variety of AM use [4].

Many treatments using AMs in children are off-label (OL). The most common reasons for this use are age, dose and indication [5]. Drug-related problems are more frequent in this age group because it is the most underserved group in clinical trials due to ethical, technical and economic impediments, thus hampering the efficacy and safety of drug therapy [6, 7]. The prevalence of OL use of medicines in hospitalized children of all age groups varies considerably (18.9-46%) [5, 8]. Some hospitals have created protocols to support evidence-based OL use to improve safety. However, many of the recommendations set out in these protocols are not applied in practice [9]. Furthermore, vulnerability associated to OL use is greater for newborns and infants.

In addition to clinical trials, it is also important to conduct drug utilization research (DUR) to determine the patterns, determinants and outcomes of the use of medicines in a real world setting [10]. However, conducting DUR in Latin America is challenging due to the complexity of the healthcare system, limited reliable data and socioeconomic disparities affecting access to care and medicines [11]. Additionally, studies on the utilization of medicines in children are further complicated by the scarcity of databases with pertinent longitudinal information, diverse procedures for recording medication use in patient records and a lack of standardization in patient data [4].

Many of the most common diseases in childhood can not be cured with older AM any longer and newer substances have been introduced, leading to possible inappropriate use, which may enhance antimicrobial resistance, considered a global threat to human health. As such investigating AM utilization is crucial mainly in developing countries where AM stewardship is poor or not implemented and protocols are feeble.

The aim of this study was therefore to describe the utilization of AMs in hospitalized children in five hospitals in Brazil, and to investigate age-related OL use in these children.

## Methods

### Study design, location and data sources

We conducted an observational study using secondary data from a multicenter prospective cohort study called “MultiCARE”. The study methodology has been reported previously [12].

The MultiCARE study was conducted in five hospitals providing medium- and high-complexity care in four of Brazil’s five regions. Three of the hospitals were university hospitals – in Rio Grande do Sul (RS), Rio de Janeiro (RJ) and Ceará (CE), with 63, 40 and 64 pediatric beds, respectively – and two were public general hospitals in the Federal District (DF) and Sergipe (SE), with 14 and 33 pediatric beds, respectively. More characteristics of the recruiting hospitals are described in Table 1.

**Table 1:** Characteristics of selected hospitals (Brazil, Multicare, 2018–2020)

	CE	DF	RJ	RS	SE	Overall
Total patients	247	147	169	284	173	1020
Median age (years)	2.8	2.8	3.1	3.3	3.1	2.8
Total number of beds	64	14	40	63	33	214
Number of days of data collection	181	183	183	184	179	182
Average occupancy rate	0.79	0.87	0.78	0.83	0.74	0.80
Patient-day	7721	2828	5710	9621	4371	30,251
Period of data colletion	Dec 1 2018 to May 31 2019	May 1 2019 to Oct 31 2019	May 1 2019 to Oct 31 2019	Aug 20 2019 to Feb 20 2020	Feb 18 2019 to Aug 16 2019	--
AM Stewardship Program	No	Yes	No	Yes	Yes	--

CE: Ceará, DF: Federal District; RJ: Rio de Janeiro; RS: Rio Grande do Sul; SE: Sergipe. (MultiCARE, 2018/2020)

The MultiCARE study data were derived from patient medical records and prescriptions. The start and end dates of the six-month collection period varied from hospital to hospital, meaning that the data were collected between 2018 and 2020. The patients participated in data collection up to completion of drug therapy or hospital discharge.

### Population and patient selection criteria

The study population comprised hospitalized pediatric patients aged 0–11 years and 11 months receiving systemic AMs for longer than 48 h. This interval is deemed best practice for antimicrobial duration before antimicrobial susceptibility testing [13]. The exclusion criteria were patients admitted to intensive care, surgical, emergency and cancer treatment units. In this regard, critically ill or hemodynamically unstable patients are more prone to problems that can affect the assessment of the use of AMs, such as changes in treatment or dose escalation. Patients who were readmitted during the study period were treated as new patients.

### Variables and medicines

The study included three groups of variables: patient characteristics and admission variables, based on data taken from patient medical records, and AM treatment characteristics, using data from prescriptions. The patient characteristics were as follows: sex, age group [14] and presence of comorbidities (disease associated with the reason for admission). The admission variables were primary diagnosis, ICU admission history during the current or previous hospital stay and length of hospital stay, calculated in days based on the date of admission and discharge.

The AM treatment characteristics were as follows: drug name and number of AMs used during hospital stay. We included medicines belonging to the Anatomical Therapeutic Chemical (ATC) classification system groups J01 (Antibacterials for systemic use) and J02 (Antimycotics for systemic use), administered orally, intravenously or intramuscularly. AMs used for a second time during the same hospital stay after stopping treatment were treated as a recurrent episode.

The results were expressed in terms of absolute and relative frequencies [15].

### Consumption analysis

Demographic and clinical characteristics were expressed as absolute and relevant frequencies.

Although defined daily dose (DDD) is considered the standard metric for assessing medicine consumption, for the purposes of this study, we used two alternative measures that are more suited to pediatric patients: DOT and LOT [16].

AM consumption was assessed using the following: (i) prescription frequency and (ii) DOT and LOT. The 1000 patient days denominator was used to allow comparisons between the hospitals and with other studies [17]. The DOT/LOT ratio was used as a proxy for the frequency of use of combination AM therapy versus monotherapy.

The results were presented through the aggregate of individual values (DOT/1000PD and LOT/1000PD), medians and interquartile ranges [15].

### Analysis of off-label use by age

AM utilization was classified as “OL use by age” when the age of a patient treated with at least one AM was younger than the age indicated on the digital drug label on the website of the national regulatory agency, ANVISA [18]. OL use was described using prevalence.

### Ethical aspects

The study used secondary data, and the identity of the participants and participating hospitals was kept confidential. The informed consent was obtained from all subjects and/or their legal guardian by signing the consent agreement in MultiCARE study [13]. The study protocol was approved by the Sergio Arouca National School of Public Health’s (ENSP/FIOCRUZ) research ethics committee (reference code 5.605.032 CAAE: 60648722.2.0000.5240).

## Results

### Study sample

The study sample consisted of 1020 patients (Table 2). Most of the children were male and aged under 2 years. Few patients had comorbidities. The hospital with the highest proportion of patients with comorbidities was the hospital in RJ. The percentage of patients with a history of ICU admission was low across all hospitals, with the hospital in SE showing the highest rate (30.6%). The hospitals in DF and RS had the highest proportion of short-stay admissions (2 to 7 days), while the hospitals in CE, RJ and SE showed the highest proportion of admissions with a hospital stay of between 8 and 14 days. Patients with chronic diseases and prolonged hospital stays were observed across all hospitals, with the highest rates being found in the hospitals in RS, CE and RJ (Table 2).

**Table 2:** Sociodemographic and clinical characteristics of patients receiving AMs by hospital

		CE	DF	RJ	RS	SE	Overall
Sex (%)	Female	117 (47.4)	58 (39.7)	82 (48.5)	117 (41.2)	83 (48.0)	457 (44.8)
	Male	130 (52.6)	88 (60.3)	87 (51.5)	167 (58.8)	90 (52.0)	562 (55.1)
	MD	0 (0.0)	1 (0.7)	0 (0.0)	0 (0.0)	0 (0.0)	1 (0.1)
Age group (%)	0-27d	3 (1.2)	3 (2.0)	2 (1.2)	9 (3.2)	1 (0.6)	18 (1.8)
	28d-23 m	133 (53.8)	69 (46.9)	76 (45.0)	135 (47.5)	89 (51.4)	502 (49.2)
	2y-6y	71 (28.7)	54 (36.7)	61 (36.1)	80 (28.2)	49 (28.3)	315 (30.9)
	6y-11y11m	40 (16.2)	21 (14.3)	30 (17.8)	60 (21.1)	34 (19.7)	185 (18.1)
Presence of comorbidities (%)	Yes	40 (16.2)	47 (32.0)	99 (58.6)	52 (18.3)	41 (23.7)	279 (27.4)
	No	207 (83.8)	100 (68.0)	70 (41.4)	232 (81.7)	132 (76.3)	741 (72.6)
History of ICU admission (%)	Yes	29 (11.7)	17 (11.6)	21 (12.4)	1 (0.4)	53 (30.6)	121 (11.9)
	No	218 (88.3)	130 (88.4)	148 (87.6)	283 (99.6)	120 (69.4)	899 (88.1)
Length of hospital stay (%)	2-7d	69 (27.9)	93 (63.3)	55 (32.5)	95 (33.5)	45 (26.0)	357 (35.0)
	8-14d	96 (38.9)	37 (25.2)	56 (33.1)	91 (32.0)	70 (40.5)	350 (34.3)
	15-30d	53 (21.5)	14 (9.5)	35 (20.7)	65 (22.9)	45 (26.0)	212 (20.8)
	31 + d	29 (11.7)	3 (2.0)	23 (13.6)	33 (11.6)	13 (7.5)	101 (9.9)
Primary diagnoses (%)		Pneumonia: 87 (35.8)	Pneumonia: 60 (40.8)	Pneumonia: 56 (33.3)	Pneumonia: 37 (13.0%)	Pneumonia: 60 (34.7%)	Pneumonia: 300 (29,6%)
		Digestive system disorders: 20 (8.2)	Skin infections: 18 (12.2)	Skin infections: 23 (13.7)	Other endocrine disorders: 28 (9.9)	Acute bronchitis or bronchiolitis: 15 (8.7)	Skin infections: 91 (9.0)
		Skin infections: 20 (8.2)	Digestive system disorders: 11 (7.5)	Other bacterial diseases: 9 (5.4)	Acute bronchitis or bronchiolitis: 21 (7.4)	Digestive system disorders: 12 (6.9)	Acute bronchitis or bronchiolitis: 53 [2, 5]

CE: Ceará, DF: Federal District; RJ: Rio de Janeiro; RS: Rio Grande do Sul; SE: Sergipe. MD: Missing data. (MultiCARE, 2018/2020)

The most frequent reason for admission was pneumonia (29.6%, *n* = 300), followed by skin infection (9%, *n* = 91) and bronchitis or bronchiolitis (5.2%, *n* = 53), across all hospitals.

### Antimicrobial consumption

Differences were observed in the number of AMs used during the hospital stay between hospitals. The frequency of use of four or more AMs during the hospital stay was highest in the hospitals in RJ and RS (Table 3).

Exposure to AMs was highest in the hospital in SE (517 DOT/1000PD, median 1.8), followed by RJ (441 DOT/1000PD, median 0.9). LOT was also highest in the hospital in SE (390 LOT/1000 PD, median 1.2), followed by RJ (311 LOT/1000 PD, median 1.2) (Table 3).

DOT/LOT ratios were high in the hospitals in RJ and CE, suggesting the possible occurrence of combination AM therapy (Table 3).

**Table 3:** AM consumption in hospitalized patients according to DOT/1000 PD and LOT/1000 PD by hospital

		CE	DF	RJ	RS	SE
Number of AMs used during hospital stay (%)	1	159 (64.4)	99 (67.3)	23 (13.6)	81 (28.5)	76 (43.9)
	2	68 (27.5)	36 (24.5)	59 (34.9)	94 (33.1)	56 (32.4)
	3	15 (6.1)	8 (5.4)	47 (27.8)	52 (18.3)	33 (19.1)
	4+	5 (2.0)	4 (2.7)	40 (23.6)	57 (20.1)	11 (4.6)
DOT/1000PD		392	278	441	404	517
DOT/1000PD median (IQR)		1.4 (1.8)	2.3 (2.3)	0.9 [1]	0.8 (1.2)	1.8 (2.1)
LOT/1000PD		276	265	311	291	390
LOT/1000PD median (IQR)		1.4 (1.4)	1.8 (1.4)	0.9 (0.6)	0.6 (0.6)	1.2 (1.2)
DOT/LOT		1.05	1.33	1.42	1.39	1.42

CE: Ceará, DF: Federal District; RJ: Rio de Janeiro; RS: Rio Grande do Sul; SE: Sergipe. IQR: interquartile range. (MultiCARE, 2018/2020)

**Fig. 1:**
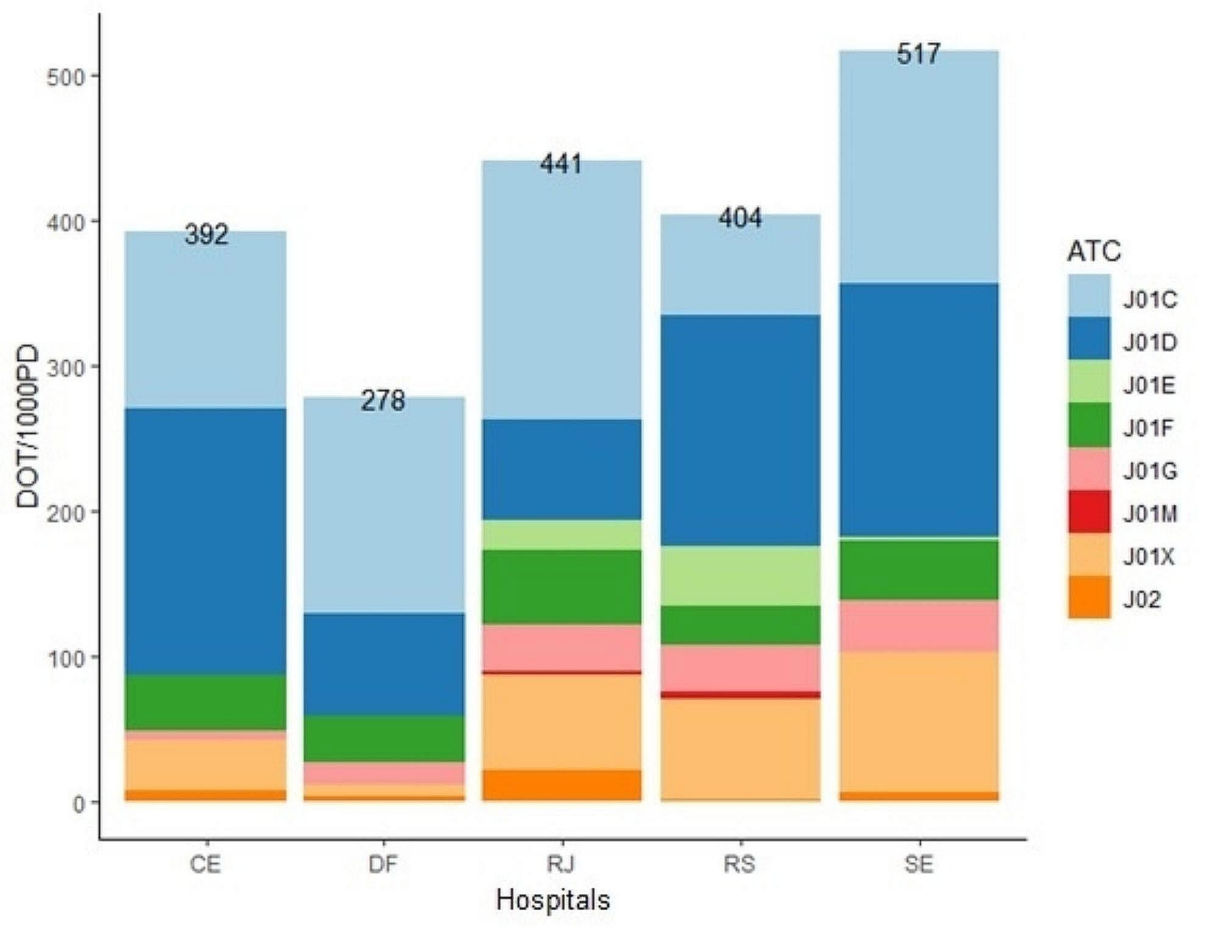
Figure 1: Consumption of AMs expressed in DOT/1000PD according to ATC codes. Brazil, 2018–2020. CE: Ceará, DF: Federal District; RJ: Rio de Janeiro; RS: Rio Grande do Sul; SE: Sergipe. ATC: Anatomical Therapeutic Chemical Classification; J01C: Beta-lactam antibacterials, penicillins; J01D: Other beta-lactam antibacterials; J01E: Sulfonamides and trimethoprim, J01F: Macrolides, lincosamides and streptogramins; J01G: Aminoglycoside antibacterials; J01M: Quinolone antibacterials; J01X: other antibacterials; J02: Antimycotics for systemic use (MultiCARE, 2018/2020)

Figure 1 presents consumption expressed in DOT/1000PD for classes in the third level of the ATC Classification. The most commonly used medicines were beta-lactam antibacterials – penicillins (J01C), cephalosporins, monobactams and carbapenems (J01D), glycopeptides, polymyxins, imidazole and nitrofuran derivatives (J01X) and macrolides, lyncosamines and streptogramins (J01F). The least used drugs were sulfonamides (J01E), aminoglycosides (J01G) and quinolones (J01M) (Fig. 1).

### Off-label use of AMs

The prevalence of OL use was highest in the hospital in RJ (23.7%) and lowest in the hospital in DF (6.8%). The overall prevalence was highest in the 28-day-23-month age group, prolonged hospital stays and use of more than one AM during the hospital (28.3%, 44.6% and 42.1%, respectively).

**Table 4:** Prevalence of AM OL use by age according to hospital and characteristics of patients

		Prevalence of OL use by age
Overall		17.6
Hospitals	CE	17.4
	DF	6.8
	RJ	23.7
	RS	20.4
	SE	16.2
Sex	Female	17.9
	Male	17.3
Age group	0-27d	11.1
	28d-23 m	28.3
	2y-6y	7.3
	6y–11y11m	6.5
Presence of comorbidities	No	17
	Yes	18.9
History of ICU admission	No	17.1
	Yes	20.7
Length of hospital stay	2-7d	12.1
	8-14d	13.4
	15-30d	20.8
	31 + d	44.6
Number of AMs used during hospital stay	1	10.7
	2	12.8
	3	28.4
	4+	42.1

CE: Ceará, DF: Federal District; RJ: Rio de Janeiro; RS: Rio Grande do Sul; SE: Sergipe (MultiCARE, 2018/2020)

The most used off-label AM was azithromycin, in both oral (*n* = 83) and parenteral (*n* = 33) formulations. OL use of oral azithromycin was highest in the hospital in CE, where the OL prescription frequency was 70.4% (data not presented). All prescriptions of parenteral azithromycin were OL in the hospitals in CE (*n* = 7), RJ (*n* = 20), RS (*n* = 1) and SE (*n* = 5) (data not presented). Other medications used off-label included piperacillin-tazobactam (*n* = 31), meropenem (*n* = 29), and ciprofloxacin (*n* = 20), among others. The complete list can be seen in the supplementary material 1.

## Discussion

The distribution of age group and sex seemed similar across the five hospitals; however, the presence of comorbidities, history of ICU admission and length of hospital stay apparently differed among them. The most common diseases were respiratory tract infections. There were wide variations in DOT/1000PD (278–517) and LOT/1000PD (265–390). The hospital in SE used more AMs than the other hospitals. The consumption of second-generation penicillins and cephalosporins was high. The prevalence of OL use of AM was higher in patients from the hospital in RJ, infants, patients who underwent prolonged hospital stays and patients receiving multiple AMs. The most commonly used off-label medicine was azithromycin, in both oral and parenteral formulations.

Studies have reported age-related differences in the pharmacokinetics and pharmacodynamics of drugs due to changes in body composition during maturation, which can affect the effectiveness of drugs [19]. There are also safety and efficacy issues related to unlicensed drug use in children [20, 21]. Although studies have shown that hospital admission rates for infections are higher in male children [22], the proportion of patients of each gender was quite similar. Furthermore, the frequency of AM use seemed similar between both sexes.

Most of the newborn and infants from our sample were admitted for respiratory tract infections (pneumonia, bronchitis or bronchiolitis) and skin infections, which is consistent with the findings of other studies with pediatric patients [23, 24]. The prevalence of comorbidities, history of ICU admission and length of hospital stay were varied and it is possible that these factors influenced medicine use [25]. However, these variables do not explain the intensive use of AMs in the hospitals since variables describing severity and AM use did not always present variation in the same direction.

There were differences in AM consumption between hospitals. The DOT/LOT ratios show that the use of multiple AMs was highest in hospitals in RS and RJ. Combination therapy is used to prevent the development of multidrug-resistant microorganisms in difficult-to-treat infections, promote synergistic interactions between active ingredients, provide a broader antibacterial spectrum and reduce the risk of mortality associated with empiric therapy [26]. However, caution should be exercised when employing this practice, as a combination of AMs can lead to severe drug‒drug interactions [27].

DOT and LOT are the most suitable measures for assessing AM consumption in pediatric patients because the defined daily dose does not provide an accurate assessment due to the small doses used in this population and the low weight of children [28]. However, DOT and LOT do not measure AM appropriateness and provide an inaccurate assessment of AM dose in patients with kidney failure [29]. In general, studies using DOT and LOT with pediatric patients show wide variations in values; however, the values found in the hospitals in SE, RJ and RS were similar to those in studies with critically ill children, suggesting intensive use of AMs [24, 30, 31]. This does not appear to be explained by the clinical condition of the patients in our study, suggesting possible overuse of AMs in these hospitals.

A study by Kreitmeyr et al. [24] in general pediatric wards in Germany reported DOT/1000PD values between 432.9 and 483.6. These values are higher than the minimum value found in the present study but show less variation. These differences may be explained by the standard of care, which varies from country to country, cultural and behavioral factors and the health care setting, including available resources, patient information, case urgency and prescriber characteristics, such as experience, level of expertise and adherence to guidelines [32]. These factors may also account for the differences in DOT/1000PD and LOT/1000PD values among the hospitals in this study. However, the results point to large variation in AM utilization and also intense use of AM, possibly related to lack of/or faulty AM stewardship. As Table 1 shows in two of the five hospitals there were no stewardship programs. Lack of adequate monitoring may lead to improper and excessive use of AM.

The most commonly used drug classes belonged to groups J01C (penicillins), J01D (cephalosporins, monobactams and carbapenems), J01X (glycopeptides, polymyxins, imidazole and nitrofuran derivatives) and J01F (macrolides, lycosamines and streptogramins). Previous studies have also reported a high frequency of use of these classes in children hospitalized for similar infections [33].

The hospital in RJ was the only hospital where antifungals were prescribed. These agents are usually prescribed for prophylaxis or the treatment of infections that generally affect neonates and immunocompromised patients [34]. Inappropriate use of antifungals is associated with the development of resistance [35]. In addition, the prevalence of OL use was highest in the hospital in RJ and lowest in the hospital in DF. It is worth highlighting that the hospital in RJ is a university hospital and provides care for children from a broader age group (0–18 years), possibly taking on patients with more severe conditions and complex care needs.

The prevalence of OL use was also high in children under two, patients who underwent prolonged hospital stays and patients receiving more than one medication. Previous studies in Brazil also found a high prevalence of OL use in patients with these characteristics [36, 37]. Prolonged hospital stay is associated with increased use of AMs due to the risk of healthcare-associated infections(55), increasing the likelihood of OL use by age.

Various studies have shown widespread OL use of systemic AMs in both outpatient [5, 20, 21] and hospitalized pediatric patients [8, 37–39]. The most commonly used classes of AMs in this population are penicillins, cephalosporins and aminoglycosides, and various reasons for OL use have been reported (age group, dose, indication, among others).

In the present study, the most commonly used off-label AM was azithromycin in both oral and parenteral formulations. Azithromycin is one of the most widely used broad-spectrum AMs for the treatment of children, especially in patients with respiratory tract infections. Azithromycin is available in parenteral formulations and is commonly administered over long periods of time at low doses in children with chronic respiratory diseases due to its immunomodulatory effect [40]. However, there is no evidence of the efficacy and safety of the use of its oral form in children under six months or its parenteral form in patients under 16 years [41], which is worrying considering the findings of the present study.

This study has several limitations. First, the data collection period was not the same in all hospitals, meaning that results could have been affected by seasonal variation in the consumption of certain AMs. Seasonal consumption is influenced by factors such as disease prevalence, seasonal epidemics, human behavior patterns and environmental conditions [42], and the study hospitals reflect diverse in-country contexts. Moreover, the results cannot be generalized because our sample was not representative. The inclusion of readmitted patients, as new patients, may burden certain results due to different severity profile of these patients. However, the units of analysis were patient medical records and prescriptions, and not individual patients. Reintroducing patients in these cases is an established procedure in DUR. Most of the studied in-patient facilities were university hospitals, in which research and teaching activities may have considerable influence on treatment strategies and may have accounted for duration and intensity of AM use, but the background of the establishments did not warrant adherence to AM protocols.

It is also important to consider the potential disadvantages of using DOT and LOT as measures of AM consumption. These measures allowed us to assess the volume of prescriptions and have some advantages over defined daily doses, since the DDD is a unit of measurement for adult populations. However, they are not able to measure the impact of the use of medicines with different spectrums of activity or the appropriateness of prescribed doses in relation to clinical protocols, among other limitations. Nevertheless these indicators have been employed in DUR for pediatric populations.

Finally, the classification of OL use was limited to age because there was insufficient information to assess AM regimen and indications, and it was not possible to estimate the probability of the occurrence of OL use using regression techniques, due to the descriptive nature of the study.

Despite its limitations, considering the overall lack of data on pediatric drug therapy in Brazil, this study provides valuable insight into the use of AM in hospitalized pediatric patients. It is crucial to acknowledge the scarcity of drug utilization studies and the lack of standardized care in Brazil. This may have implications for the rational use of AM, particularly concerning the choice of pharmacological classes and the quantities of administered medicines. These factors should be taken into account when evaluating the appropriate use of AM for these patients, as well as for pediatric patients in the country, in other Latin American countries, and in similar contexts. Understanding medication utilization in different countries and contexts is essential for achieving greater rationality in drug use especially for AM.

## Conclusion

This study described the characteristics and use of AMs in children admitted to pediatric wards in five hospitals in Brazil. The results reveal intensive use of AMs and wide variations in DOT and LOT values. The use of AMs in hospitalized pediatric patients is common and varies from hospital to hospital, influenced mainly by the specific characteristics of each setting and the prescribing standards adopted by pediatricians. Further research is needed to assess the impact of certain types of treatment strategies on the use of medicines and to investigate the risk factors associated with AM use, including OL use of AMs in hospitalized children.

The results highlight the need for more careful monitoring of AM use and the development of guidelines on the prescription of AMs in this population, focusing on factors such as age group, dosage regimen and indication. Hopefully these results will encourage further research on this topic and the implementation of more rigorous policies to ensure the rational use of AMs among hospitalized pediatric patients in Brazil. By advancing our understanding of prescribing standards and the utilization of medicines in this population, we can improve the quality of health care, reduce antimicrobial resistance and improve pediatric patient outcomes.

### Electronic supplementary material

Below is the link to the electronic supplementary material.

**Supplementary Material 1: Table S1.** Number of off-label prescriptions of antimicrobials and off-label prescription frequency

## Data Availability

The datasets supporting the conclusions of this research article are available by emailing the corresponding author.

## Abbreviations

AM: antimicrobial
ATC: Anatomical Therapeutic Chemical
CE: Ceará
DDD: Defined daily dose
DF: Distrito Federal
DOT: Days of therapy
DUR: drug utilization research
ICU: Intensive care unit
LOT: Length of therapy
OL: Off-label use
RJ: Rio de Janeiro
RS: Rio Grande do Sul
SE: Sergipe
