# Supplementary material for: Use of antimicrobials in pediatric wards of five Brazilian hospitals
Source: BMC Pediatr. 2024 Mar 13;24:177. doi: 10.1186/s12887-024-04655-9 (PMC10936065; doi:10.1186/s12887-024-04655-9)
Supplement: Supplementary file 1 — Supplementary Material 1: Table S1. Number of off-label prescriptions of antimicrobials and off-label prescription frequency [file 12887_2024_4655_MOESM1_ESM.docx]

**SUPPLEMENTARY MATERIAL**

**Table S1:** Number of off-label prescriptions of antimicrobials and off-label prescription frequency.

| Antimicrobial | Age indication (label) | OL prescriptions (%) |
| --- | --- | --- |
| Azithromycin (O) | over 6 months | 83 (64.8) |
| Azithromycin (P) | over 16 years | 33 (100) |
| Piperacillin/Tazobactam (P) | over 2 years | 31 (86.1) |
| Meropenem (P) | over 3 months | 29 (45.3) |
| Ciprofloxacin (P, O) | over 5 years | 20 (100) |
| Trimethoprim-sulfamethoxazole (P, O) | over 6 weeks | 14 (24.6) |
| Clindamycin (P) | over 6 months | 1 (2.2) |
| Clarithromycin (O) | over 1 month | 1 (25) |
| Doxycycline (O) | over 8 years | 1 (100) |
| Nitrofurantoin (O) | over 16 years | 1 (100) |

P: parenteral; O: oral (MultiCARE, 2018/2020).
